# 

**Published:** 1896-11

**Authors:** 


### CONTENTS—NOVEMBER, 1896.—VOL. XII., NO. 5.

ORIGINAL CONTRIBUTIONS—

Would Asexualization of Chronic Criminals, Sexual Perverts and Hereditary Defectives Benefit Society and Elevate the Human Race? E. Stuver, M. D. . . . . . . . . . . . 225
A Case of Protracted Labor. J. N. Upshur, M. D. . . . . . . . . . . . . . . . . . . 231
Conservatism in Gun-Shot Wounds of the Abdomen. J. M. Fry, M. D . . . . . . . . 235
The Lunatic Asylums and the Medical Colleges. F. S. White, M. D. . . . . . . . . 238
Imperforate Hyman. J. Llewellyn Jones . . . . . . . . . . . . . . . . . . . . . 239

CORRESPONDENCE—

Too Good to Keep—Differential Diagnosis . . . . . . . . . . . . . . . . . . . . 240
The Recognition of Illegal Practice. . . . . . . . . . . . . . . . . . . . . . . 242

SOCIETY NOTES—

Southern Surgical and Gynecological Association. . . . . . . . . . . . . . . . . 243
West Texas District Medical Society. . . . . . . . . . . . . . . . . . . . . . . 245

ABSTRACTS AND SELECTIONS—

Is There a Typho-Malarial Fever? A Century of Vaccination. What Sanitation Has Done for Life? Local Treatment of Diphtheria. And other Items. . . . . . . . . . 246–258

EDITORIAL—

An Embryo State Training School. . . . . . . . . . . . . . . . . . . . . . . . . 259
Dr. J. B. Hamilton . . . . . . . . . . . . . . . . . . . . . . . . . . . . . . . 261
Hypnotism as She is Taught in Chicago . . . . . . . . . . . . . . . . . . . . . 263
The Atlanta Freak . . . . . . . . . . . . . . . . . . . . . . . . . . . . . . . 266
A Soft Thing. . . . . . . . . . . . . . . . . . . . . . . . . . . . . . . . . . 267
Don't Monkey with the Buz-Saw. . . . . . . . . . . . . . . . . . . . . . . . . . 267
"A Cumulative Action of Alcohol" . . . . . . . . . . . . . . . . . . . . . . . . 268
A Medical Sensation . . . . . . . . . . . . . . . . . . . . . . . . . . . . . . 269
The Song of the Peripatetic. . . . . . . . . . . . . . . . . . . . . . . . . . . 269
The International and Great Northern. . . . . . . . . . . . . . . . . . . . . . 271
The Land of Montezuma . . . . . . . . . . . . . . . . . . . . . . . . . . . . . 272
Transactions Texas State Medical Association. . . . . . . . . . . . . . . . . . 274

MEDICAL NEWS AND MISCELLANY. . . . . . . . . . . . . . . . . . . . . . . . . . 274–280

Journal's Own Page . . . . . . . . . . . . . . . . . . . . . . . . . . . . . . . 280
Journal's Exchange Bureau . . . . . . . . . . . . . . . . . . . . . . . . . . . 280

PUBLISHRRS' NOTES . . . . . . . . . . . . . . . . . . . . . . . . . . . . . . . 282–288

